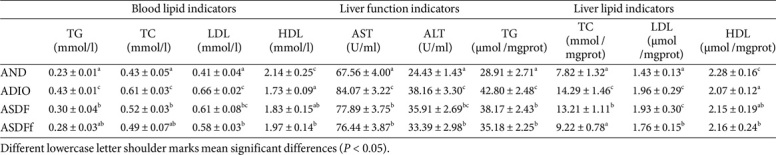# Protective Effects of Ginseng Soluble Dietary Fiber and Its Fecal Microbiota Extract on Antibiotic-Induced Gut Dysbiosis Obese Mice

**DOI:** 10.4014/jmb.2502.02013

**Published:** 2025-07-14

**Authors:** Luran Yang, Mei Hua, Da Li, Fan Li, Yuguang He, Xinyu Miao, Mubai Sun, Honghong Niu, Fenghao An, Jing Wang, Min Yang, Jinyuan Lu, Hongyan Xu, Jinghui Wang

**Affiliations:** 1 Agronomy of Food Science and Engineering, Yanbian University, Yanji 133002, Jilin, P.R. China; 2 Institute of Agro-food Technology, Jilin Academy of Agricultural Science (Northeast Agricultural Research Center of China), Changchun 130033, Jilin, P.R. China; 3 School of Life Science, Northeast Normal University, Changchun 130033, Jilin, P.R. China; 4 School of Life Science, Jilin Normal University, Siping 136000, Jilin, P.R. China

**Keywords:** Ginseng soluble dietary fiber, fecal microbiota transplantation, antibiotic-induced gut dysbiosis, obese mice, LPS/TLR4/MyD88/NF-κB pathway, intestinal flora

## Abstract

Prolonged or improper antibiotic use may increase the risk of obesity. Ginseng soluble dietary fiber (G-SDF) has been shown to inhibit obesity and promote the growth of intestinal probiotics. However, its role in antibiotic-induced gut dysbiosis obese mice (ADIO) remains unclear, and this study aimed to elucidate this role. The results indicated that G-SDF and its fecal microbiota extract (SDFfbs) significantly reduced body weight, insulin resistance, hepatic fat accumulation, abnormal blood and liver glucose-lipid metabolism, oxidative stress, and immune-inflammatory responses in ADIO mice. G-SDF and SDFfbs also inhibited the LPS/TLR4/MyD88/NF-κB signaling pathway, restored the expression of the gut barrier proteins Occludin and Claudin1, and protected against intestinal damage in ADIO mice. In particular, G-SDF and SDFfbs significantly increased the abundance of Firmicutes and Bacteroidetes and decreased the abundance of harmful *Escherichia* and *Streptococcus*. Additionally, they promoted the growth of beneficial bacteria, such as *Enterococcus*, *Lactobacillus*, *Bifidobacterium*, *Parabacteroides*, and *Akkermansia*, and these microbial shifts correlated with significant improvements in metabolic indicators in ADIO mice. Notably, SDFfbs can replicate the efficacy of SDF and has even shown stronger effects than the latter. In summary, this study demonstrated that G-SDF and SDFfbs effectively mitigate the double damage caused by obesity and antibiotic exposure by modulating the LPS/TLR4/MyD88/NF-κB pathway, protecting the intestinal barrier, and restoring the gut microbiota balance. These findings provide an important theoretical basis for the use of G-SDF and SDFfbs as fat-reducing and antibiotic-resistant ingredients in health foods.

## Introduction

Obesity is a chronic metabolic disease influenced by various factors, including genetics, environmental conditions, endocrine dysregulation, inflammation, and the composition of the gut microbiota [1]. According to the World Obesity Federation (WOF) in the 6th edition of the World Obesity Atlas 2023 [2], an estimated 3.3 billion adults worldwide will be affected by overweight and obesity by 2035. Antibiotics are widely used in clinical practice. However, prolonged or inappropriate antibiotic use may increasing the potential risk of obesity [3, 4]. Studies have shown that antibiotic treatment reduces PPAR-γ receptor activity in the intestinal epithelial cells of obese mice, potentially exacerbating obesity progression [5]. Conversely, another study suggested that antibiotics have limited metabolic effects in healthy adolescents and obese adults [6, 7]. These studies suggest that the impact of antibiotics on obesity may vary depending on factors such as antibiotic type, dosage, medication cycle, and individual genetics. Despite controversy, increasing evidence suggests an association between antibiotic use and obesity development. Therefore, further research is needed to explore the effects of antibiotics on the gut microbiota and metabolic processes in obese individuals and to investigate dietary components that could replace antibiotics or mitigate their effects to aid in obesity prevention and treatment.

Recognized as a prominent medicinal food, ginseng (*Panax ginseng* C.A. Meyer) and its active compounds (*e.g.*, ginsenosides) have been repeatedly shown to promote weight loss and improve abnormal glycolipid metabolism in obese animal models [8, 9]. Additionally, ginseng has shown potential as an antibiotic alternative, which can possibly reduce the antibiotic-associated side effects. A study has shown that American ginseng dietary fiber enhances the integrity of the intestinal mucosal barrier and immunity in immunosuppressed mice by regulating the TLR4/NF-κB signaling pathway, which is a key mechanism in anti-inflammatory and immunomodulatory responses [9]. Our previous studies demonstrated that ginseng-soluble dietary fiber (G-SDF) improves glucose-lipid metabolism (particularly triglyceride levels), increases satiety and energy balance, and promotes the growth of probiotics (*e.g.*, *Lactobacillus* and *Bifidobacterium*) in rats [10-12]. These findings suggest that G-SDF may be considered a potential health ingredient for weight loss and could be incorporated into diets interventions involving antibiotics. In summary, this study aimed to reveal the protective effects of G-SDF and its fecal microbiota extract (SDFfbs) on glucose–lipid metabolism, the inflammatory response and damage to the gut microbiota in obese mice under the interference of complex antibiotics and preliminarily elucidates the weight loss mechanisms via the gut‒liver axis.

## Materials and Methods

### Animals and Ethical Approval

Four-week-old, specific pathogen-free (SPF) male C57BL/6J mice (*n* = 24) were obtained from Liaoning Changsheng Biotechnology Company Limited (China) with Animal Production License No. SCXK 2020-0001 and were housed at the animal experimental apparatus of Institute of Agro-food Technology, Jilin Academy of Agricultural Science. Upon arrival, animals were given a 7-day acclimatization period and were maintained at a constant temperature of 20–24°C and relative humidity of 40%–60%. The animals were kept on a 12 h day/night circadian rhythm cycle, and food and water were supplied ad libitum. To eliminate confounders, the animals were housed in the same cage. By the conclusion of the acclimatization period, the animals exhibited similar body weights (16.23 ± 0.95 g), and were randomly assigned to each treatment group. According to animal management and welfare, in order to minimize the number of mice subjected to the experiment, and the size of the mouse cage was taken into consideration to prevent overcrowding and poor-living conditions for each group of mice, the sample size was set at 6 mice per group. All animal experiments were conducted in compliance with the guidelines of the Ethics Committee on Laboratory Animal Management and Welfare at the Academy of Agricultural Sciences of Jilin Province.

Mouse growth and breeding normal diet (ND, Product No. 1016706476803973120) and high-fat diet (HFD, Product No. D12492) were obtained from Keao Xieli Feed Co., Ltd. (China).

### Preparation of G-SDF

Following the method of Hua *et al*. [10] for extracting the G-SDF, the product yield of G-SDF in ginseng residue was 15.46%.

### Preparation of SDFfbs

In the preliminary experiment, 4-week-old SPF-grade male C57BL/6J mice (*n* = 12) were used to establish an obesity model (DIO) by feeding HFD for 8 weeks. Then with the same feed, except for the DIO group (randomly assigned according to the body weight, *n* = 6), the treatment group (randomly assigned according to the body weight, *n* = 6) received continuous gavage administration of G-SDF (1,200 mg/kg, 200 μl per mouse) for 6 weeks, with feces collected aseptically and stored at -80°C. The collected feces were prepared as a 10% (v/w) suspension in sterile saline, vortexed, thoroughly mixed, at 4°C, 1,500 rpm, centrifuge for 5 min, take the supernatant. The supernatant bacterial solution was collected and prepared as SDFfbs (containing at least 1.0 × 10^7^ CFU/ml viable bacteria) and stored at 4°C. Fresh SDFfbs preparations were made every 2 days. Chemical composition of G-SDF and viable bacteria count of SDFfbs were shown in Table S1.

### Experimental Design

**Fig. 1:**
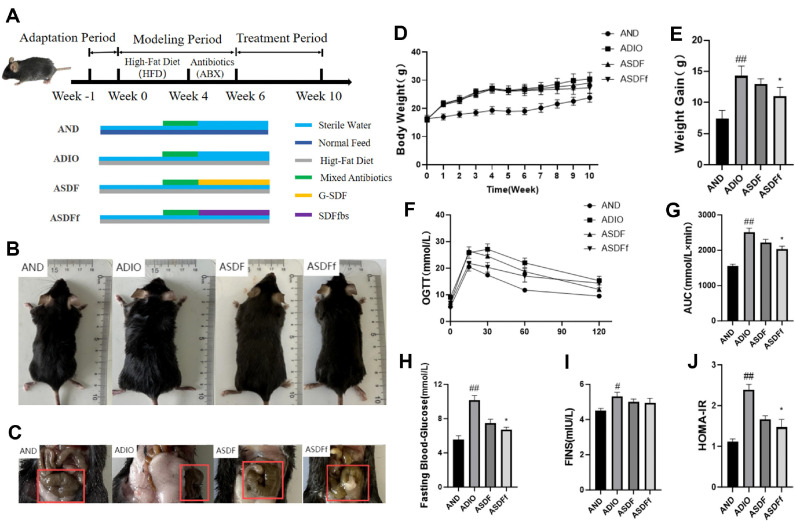
Experimental design (A) mouse appearance (B) cecum (C) body weight (D-E) blood sugar and insulin levels (F-J) (*n* = 6). Compared with the AND group, ^#^*P* < 0.05, ^##^*P* < 0.01. Compared with the ADIO group, *P < 0.05, **P < 0.01. The same below.

The experimental design is illustrated in Figure 1A. After 7 days adaptation period, C57BL/6J mice (*n* = 24) were randomly divided into 4 groups, with 6 mice in each group placed in a cage: AND, ADIO, ASDF and ASDFf. To eliminate confounders, the animals were housed in the same environmental conditions, both gavage and feeding were performed by the same experimenter at a fixed time (10:00 a.m. to 11:00 a.m.). Three groups (ADIO, ASDF, and ASDFf) received HFD, while the control group (AND) received ND throughout weeks 1-10th. During weeks 4-6th, mice in each group received daily gavage with a combination of antibiotics (ABX: penicillin 1 g/l, neomycin 1 g/l, metronidazole 1 g/l, and vancomycin 0.5 g/l). Antibiotic treatment was discontinued during weeks 6-10. According to the recommended daily intake of dietary fiber (DF) for the human body (20-25 g DF/60 kg body weight/day, 7-8 g SDF/60 kg/day) [13] and the results of previous studies for G-SDF and fecal microbiota transplantation (FMT) [8, 11, 12, 14], the ASDF group received G-SDF gavage (1,200 mg/kg), while the ASDFf group received 200 μl SDFfbs (≥1.0 × 10^7^ CFU/ml viable bacteria) by oral gavage daily as FMT. Meanwhile, both ND and HFD were provided in each group of cages, allowing the animals to choose freely. Growth parameters, including body weight, food intake, and water intake, were recorded weekly. At the end of the study, fecal samples were rapidly collected using sterile cryopreserved tube in a sterile operating station and stored at -80°C for subsequent analysis.

### Measurement of Fasting Blood Glucose and Lipid Indices

One week before the experiment concluded, following a 12-h fast, blood samples were collected from the tail vein to measure fasting blood glucose (FBG) levels. Three days after FBG measurement, an oral glucose tolerance test (OGTT) was conducted. After an 8-h fast, a 10% glucose solution (2 g/kg) was administered via gavage, and blood glucose levels were measured at 15, 30, 60, and 120 min post-gavage using a glucose meter (Roche ACCU-CHEK, Germany). The area under the curve (AUC) of the OGTT was subsequently calculated [15].

One day before the experiment ended, mice were fasted and deprived of water for over 12 h. Blood samples were subsequently collected from the retro-orbital sinus anesthetized with tribromoethanol (0.2 ml/10 g) and left to stand at 37°C for 1 h. The blood was then centrifuged at 2,000 rpm for 10 min, and serum was extracted from the supernatant. Serum insulin levels were measured, and the insulin resistant (HOMA-IR) index was calculated using the following formula:

HOMA-IR = fasting insulin (mU/l) × fasting glucose (mM) / 22.5

Serum superoxide dismutase (SOD), malondialdehyde (MDA), total cholesterol (TC), triglycerides (TG), high-density lipoprotein cholesterol (HDL-C), low-density lipoprotein cholesterol (LDL-C), aspartate aminotransferase (AST), and alanine aminotransferase (ALT) were measured with commercial assay kits (JianCheng Technology Co., Ltd, China) according to the manufacturer’s instructions. Serum immunoglobulin G (IgG, RD-RX20509J), immunoglobulin A (IgA, RD-RX20506J), immunoglobulin M (IgM, RD-RX20513J), tumor necrosis factor-α (TNF-α, RD-RX20852J), and interleukin-10 (IL-10, RD-RX20162J) levels were quantified using mouse ELISA kits (RuiDaHengHui, China). Lipopolysaccharide (LPS, RD-RX20839J), fasting insulin (FINS, RD-RX20778J), adiponectin (ADPN, RD-RX20844J), leptin (LEP, RD-RX20624J), and glucagon-like peptide-1 (GLP-1, RD-RX20787J) levels were also quantified using the ELISA kits (RuiDaHengHui). Total protein concentrations were determined using a BCA protein assay kit (Solarbio, PC0020, China).

### Histopathological Staining

After the blood collection, mice were dissected, the liver, colon, epididymal white adipose tissue (eWAT), inguinal white adipose tissue (iWAT), and brown adipose tissue (BAT) were rapidly excised on ice. Portions of the tissues were immediately fixed in 4% paraformaldehyde, while the remaining colon tissues and cecal contents were stored at -80°C. Colon samples were fixed in 4% paraformaldehyde for 48 h, rinsed in running water, dehydrated through a graded ethanol series, cleared in xylene, permeabilized with wax, embedded, and deparaffinized for sectioning. Sections were stained with Oil Red O and Hematoxylin and Eosin (HE) and examined under a microscope for tissue structure, with images subsequently captured.

### Western Blot Analysis

Inflammatory and barrier-associated proteins, including Toll-Like Receptor 4 (TLR4, Proteintech, 66350-1-lg, China), Nuclear Factor Kappa-B p65 (NF-κB p65, Proteintech, 10745-1-AP, China), Myeloid Differentiation Protein-88 (MyD88, Proteintech, 23230-1-AP, China), Occludin (Proteintech, 27260-1-AP, China) and Claudin1 (Proteintech, 13050-1-AP, China) were detected in colon tissue using Western blot analysis. Colon tissue samples were incubated in RIPA buffer (Proteintech, PR20036, China) containing 1 mM PMSF (Servicebio, G2008-1ML, China) on ice for 30 min, followed by centrifugation at 12,000 rpm for 10 min. The supernatant was collected, and protein concentration was measured using a BCA protein assay kit (Solarbio, PC0020). Protein extract (5 μg/μl) was mixed with loading buffer (Servicebio, G2075-1ML, China) and heated in a boiling water bath for 10 min. Denatured proteins were separated by SDS-PAGE and transferred to a PVDF membrane (Millipore, ISEQ00010, Britain). The membrane was blocked with a protein-free blocking solution (Servicebio, G2052-500ML, China) for 5 min at room temperature, incubated overnight at 4°C with the primary antibody, and subsequently incubated with a horseradish peroxidase-conjugated secondary antibody. Protein bands were visualized on a chemiluminescence imaging system (Servicebio, SCG-W3000, China). Band grayscale values were quantified using ImageJ software and normalized to β-actin (Servicebio, GB15003-100, China).

### Analysis of 16S rRNA Gene in Intestinal Flora

Total genomic DNA from enteric bacteria was extracted using the QIAamp Fast DNA Extraction Kit (Qiagen, 51604, Germany), following the manufacturer's instructions. Sample concentration and purity were assessed using a NanoDrop 2000c spectrophotometer (Thermo Fisher Scientific, USA) and verified by agarose gel electrophoresis. The V3-V4 region of the bacterial 16S rRNA gene was amplified by PCR using a forward primer (5'-ACTCCTACGGGGAGGCAGCA-3') and a reverse primer (5'-TCGGACTACHVGGGTWTCTAAT-3'). The PCR reaction mixture (20 μl) contained 5×TransStart FastPfu PCR Buffer (4 μl), dNTPs (2.5 mmol/l, 2 μl), TransStart FastPfu DNA Polymerase (2.5 U/μl, 0.5 μl), forward and reverse primers (10 μmol/l, 0.5 μl each), template DNA (2 μl), and ddH_2_O (10.5 μl). PCR cycling conditions were as follows: initial denaturation at 94°C for 4 min; 30 cycles of 94°C for 30 sec, 50°C for 30 sec, and 72°C for 30 sec; final extension at 72°C for 5 min. Amplified products were quantified using the PicoGreen dsDNA Quantification Kit (Invitrogen, USA).

Pooled amplified sequences were subjected to paired-end sequencing on the Illumina NovaSeq 6000 System with the MiSeq Reagent Kit v3 (Shanghai Parsonage Biotechnology Co., China). Sequencing data were processed through the Quantitative Insights Into Microbial Ecology (QIIME 2.0) pipeline. Quality control steps, including de-priming, quality filtering, denoising, splicing, and chimera removal, were performed with the DADA2 method. Denoised high-quality sequences generated by DADA2 were clustered into operational taxonomic units (OTUs) at a 97% identity threshold using UCLUST. OTUs with an abundance below 0.001% of total sequences were discarded from all samples. Representative sequences were selected from the OTUs using default parameters and taxonomically classified using the BLAST method in the Greengenes database on a rank-by-rank basis.

### Statistical Analysis

Graphpad Prism 9.0 was used for drawing, ImageJ was used for area calculation and IBM SPSS Statistics 27 was used for statistical analysis. Before performing ANOVA, all data were quality-checked using boxplot analysis to guarantee they followed normal distributions. The one-way ANOVA method was used to evaluate the treatment effects of each outcome, and LSD method was selected for post hoc analysis to compare the means between groups. Unless otherwise specified, all results are expressed as means ± standard deviation (*n* = 6). *P*-value of <0.05 was considered statistically significant (*), while *P*-value of <0.01 was considered highly significant (**). No adverse events during the experiment (*e.g.*, attacked or bitten, drug allergies, and abnormal deaths). So that no animals or data points were excluded from the study during the analysis process.

## Results

### Effect of G-SDF and SDFfbs on Basal Indices

As shown in Figure 1, ABX interference did not eliminate obesity symptoms in ADIO mice (Fig. 1B). However, both the ASDF and ASDFf groups demonstrated significant reductions in body weight, adiposity coefficient, FBG, OGTT and HOMA-IR indices (*P* < 0.01), along with a significant increase in INS levels (*P* < 0.05, Fig. 1D-1J). Additionally, ABX caused significant edema in the cecum and colon of ADIO group, while the ASDF and ASDFf groups showed partial improvement (Fig. 1C). Compared to the ADIO group, the ASDFf group treated with FMT showed significantly lower weight gain, iWAT, and eWAT indices (*P* < 0.05) and a higher BAT index (*P* <0.05) (Table S2).

### Effects of G-SDF and SDFfbs on Blood Lipid and Liver Fat

As shown in Table 1, under the ABX interference, serum and liver levels of TG, TC, and LDL of the ADIO group were significantly elevated (*P* < 0.05), while HDL levels were significantly reduced (*P* < 0.05) compared to those of the AND group. Compared to the ADIO group, the ASDF and ASDFf groups showed significantly reduced TG, TC, LDL levels and increased HDL levels (*P* < 0.05), and the ASDFf group exhibited significantly improvement of Liver TC and LDL levels (*P* < 0.05) than that of the ASDF group (Table 1).

### Effect of G-SDF and SDFfbs on Histopathology

**Fig. 2:**
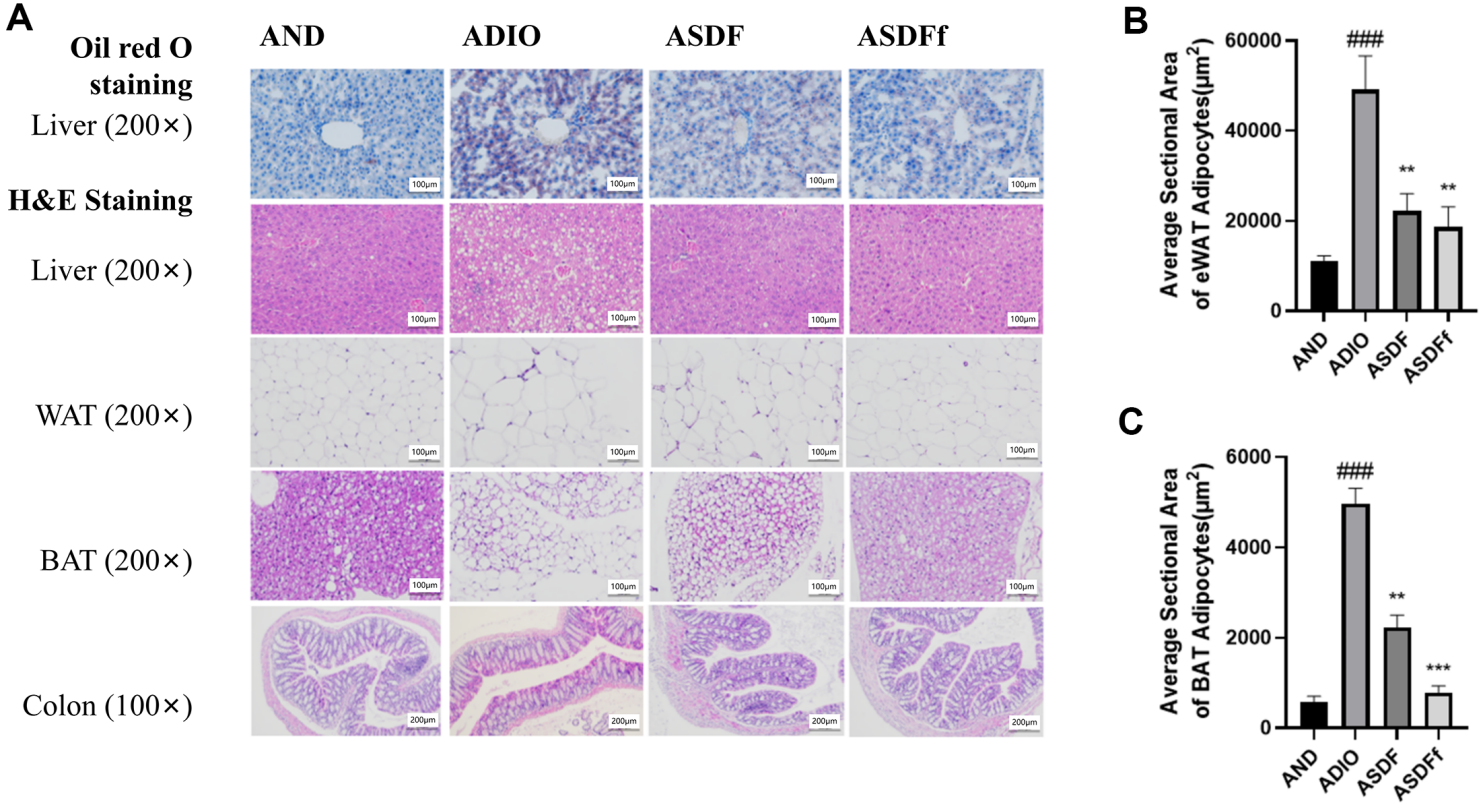
Mouse liver, WAT, BAT, and colon Histopathologic sections (A) and adipocyte surface area analysis (B-C).

As shown in Figure 2A, Oil Red O staining confirmed substantial fat droplet accumulation in the ADIO Mice liver, which was significantly reduced by the G-SDF and SDFfbs treatments. HE staining further revealed that lipid accumulation and the surface area of WAT and BAT cells in the ADIO group were significantly larger than those of the other three groups (*P* < 0.05, Fig. 2B and 2C). Notably, ABX caused severe damage to the colonic villous structure in the ADIO group, while the G-SDF and FMT treatments in ASDF and ASDFf groups effectively protected against the colon structural damage caused by antibiotics and HFD.

### Effects of G-SDF and SDFfbs on Oxidative Stress, Inflammation, and Immune Levels

**Fig. 3:**
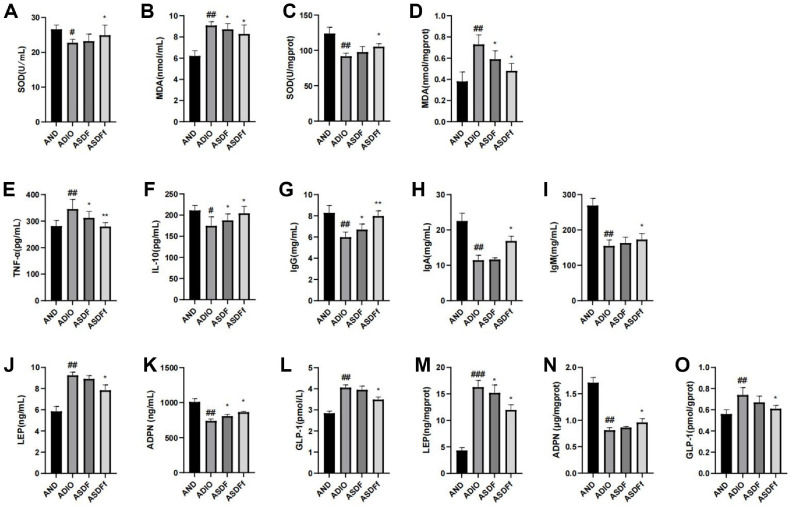
Detection of antioxidant (A-D), inflammatory (E-F), immune (G-I), and lipid metabolism factor levels (J-O) in the serum and tissues of mice.

As shown in Figure 3, the ADIO group exhibited significantly lower serum and liver SOD activity and higher MDA content compared to the AND group. These oxidative stress indicators were significantly improved in the ASDF and ASDFf groups (Fig. 3A-3D). Additionally, serum TNF-α level was significantly elevated (*P* <0.05) and the IL-10 level was significantly reduced (*P* < 0.05) in the ADIO group compared to the AND group. These inflammatory indicators were reversed by G-SDF and FMT treatments in ASDF and ASDFf groups (Fig. 3E and 3F). Furthermore, the serum IgG, IgA, and IgM levels were significantly reduced in the ADIO group. Following the G-SDF and FMT treatments, serum IgG, IgA, and IgM levels improved in the ASDF and ASDFf groups, and the ASDFf group showing the most pronounced effect (*P* < 0.05, Fig. 3G-3I).

### Effect of G-SDF and SDFfbs on the Lipid Metabolism Factors

As shown in Figure 3, using antibiotics alone did not normalize the lipid metabolism levels in ADIO mice. Compared to the AND group, the ADIO group exhibited significantly elevated LEP and GLP-1 levels (*P* < 0.01) and declined ADPN levels (*P* < 0.01). In contrast, the ASDF and ASDFf groups improved the levels of these factors to varying degrees, and the ASDFf group showing the most pronounced effect (*P* < 0.05, Fig. 3J-3O).

### Effects of G-SDF and SDFfbs on Intestinal Barrier Proteins and LPS/TLR4/MyD88/NF-κB Pathway

**Fig. 4:**
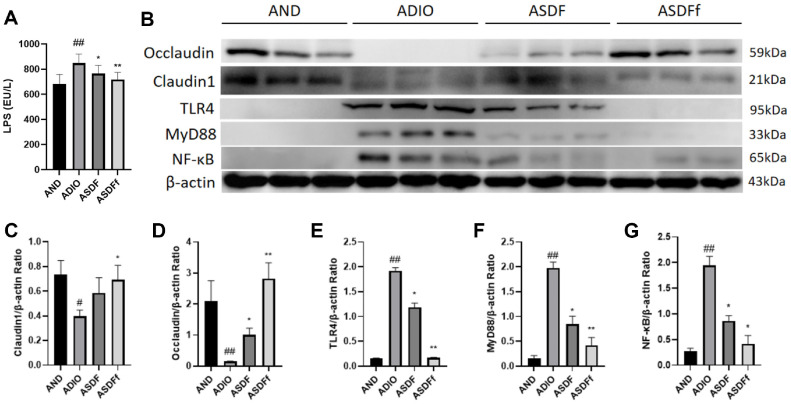
Serum LPS level (A) Immunoblot images and densitometric quantitative analysis (B-G) of Occludin, Claudin1, TLR4, MyD88, and NF-κB in gut-disordered obese mouse colon (*n* = 3).

As shown in Fig. 4, antibiotics exacerbated damage to the intestinal barrier in ADIO mice. Compared to the AND group, the LPS level was significantly elevated (*P* < 0.01), and the expression levels of barrier proteins Occludin and Claudin1 were significantly reduced in the ADIO group (*P* < 0.01). Following the G-SDF and FMT interventions, the LPS, Occludin and Claudin1 levels were reversed significantly in the ASDF and ASDFf groups, and the ASDFf group shown a more significant effect on LPS, Occludin and Claudin1 levels (*P* < 0.01 or *P* < 0.05) than the ASDF group (*P* < 0.05 or no significance, Fig. 4A-4D).

Additionally, the proteins expression of TLR4, MyD88, and NF-κB p65 in the colonic tissues of ADIO mice were significantly elevated compared to the AND group (*P* < 0.05). Following the G-SDF and SDFfbs interventions, the expression levels of these proteins were significantly reduced in the ASDF and ASDFf groups, indicating inhibition of the LPS/TLR4/MyD88/NF-κB signaling pathway (Fig. 4B), and the inhibitory effect on the expression levels of TLR4 and MyD88 proteins of the ASDFf group (*P* < 0.01) was more significant than that in the ASDF group (*P* < 0.05, Fig. 4E and 4F).

### Effect of G-SDF and SDFfbs on the Intestinal Flora Structure

**Fig. 5:**
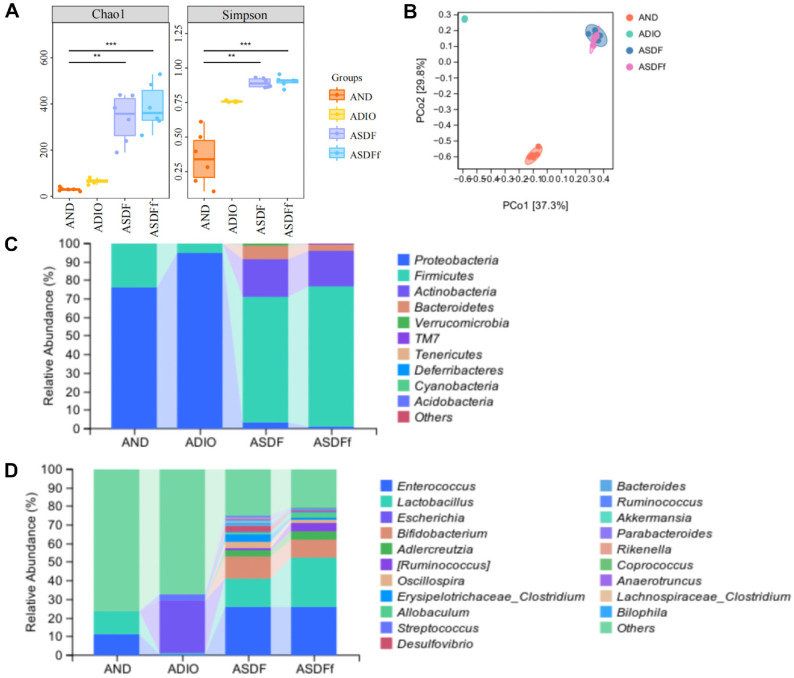
Effect of G-SDF and SDFfbs on intestinal flora α-diversity (A) Chao1 and Simpson index. **P < 0.01, ***P < 0.001, AND vs ASDF or ASDFf groups), β-diversity (**B**) PCoA based on Bray-Crutis distance) and abundance at the phylum (**C**) and the genus levels (**D**) in ADIO mice (*n* = 6).

To investigate the effect of ABX interference on intestinal flora structure in obese mice, we performed high-throughput sequencing of bacterial 16S rRNA using the Illumina MiSeq platform. As shown in Fig. 5A, ABX interference caused decreases in the Chao1 and Simpson indices in the AND and ADIO groups, especially in the ADIO group. In contrast, these diversity indices were significantly higher in the ASDF and ASDFf groups. PCoA analysis of β-diversity revealed significant structural differences in intestinal flora among all the groups (Fig. 5B). The ADIO group was clearly separated from the AND group, whereas the ASDF and ASDFf groups clustered together closely, suggesting that G-SDF can specifically modulate certain intestinal flora and that SDFfbs can replicate the microbiota-regulating effects of G-SDF.

**Fig. 6:**
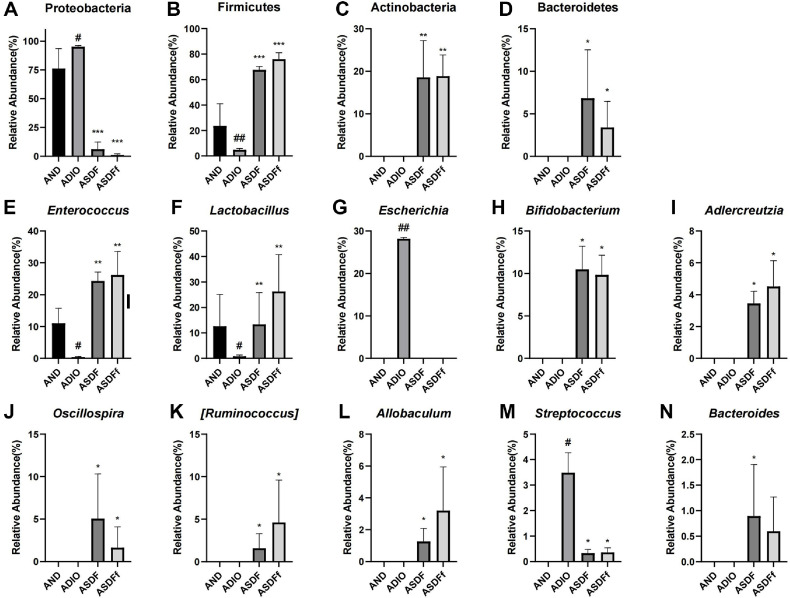
Abundance statistics of intestinal flora with significant differences at the phylum (A-D) and the genus levels (E-N) in ADIO mice (*n* = 6). Compared with the AND group, ^#^*P* < 0.05, ^##^*P* < 0.01. Compared with the ADIO group, *P < 0.05, **P < 0.01, ***P < 0.001.

These findings were confirmed by abundance analysis of intestinal flora at the phylum and genus levels. At the phylum level, ABX interference led to Proteobacteria and Firmicutes becoming the dominant phyla in the AND and ADIO groups, while Proteobacteria occupying a particularly large proportion in the ADIO group. In contrast, the abundance of Actinobacteria and Bacteroidetes was significantly higher in the ASDF and ASDFf groups, along with a significant increase in Firmicutes (*P* < 0.05, Figs. 5C and 6A-6D).

At the genus level, the primary bacteria in the AND group were *Lactobacillus* and *Enterococcus*, whereas the major bacteria in the ADIO group included *Escherichia*, *Streptococcus*, and *Enterococcus*. The elevated abundance of *Escherichia* and *Streptococcus* suggested that the intestinal flora structure of ADIO mice is more susceptible to disrupted by ABX (*P* < 0.05, Figs. 5D and 6G-6M). The abundance of *Enterococcus* and *Lactobacillus* were significantly higher in the ASDF and ASDFf groups compared to the ADIO group (*P* < 0.01, Figs. 5D and 6E-6F). Additionally, the abundance of *Bifidobacterium*, *Adlercreutzia*, *Oscillospira*, [*Ruminococcus*], *Allobaculum*, and *Bacteroides* were significantly higher in the ASDF and ASDFf groups compared to the ADIO group (*P* < 0.05, Figs. 5D, 6H-6L, and 6N). The significantly different intestinal flora with abundance below 1% at the genus level were shown in Figure S1.

### Effect of G-SDF and SDFfbs on Intestinal Marker Species

**Fig. 7:**
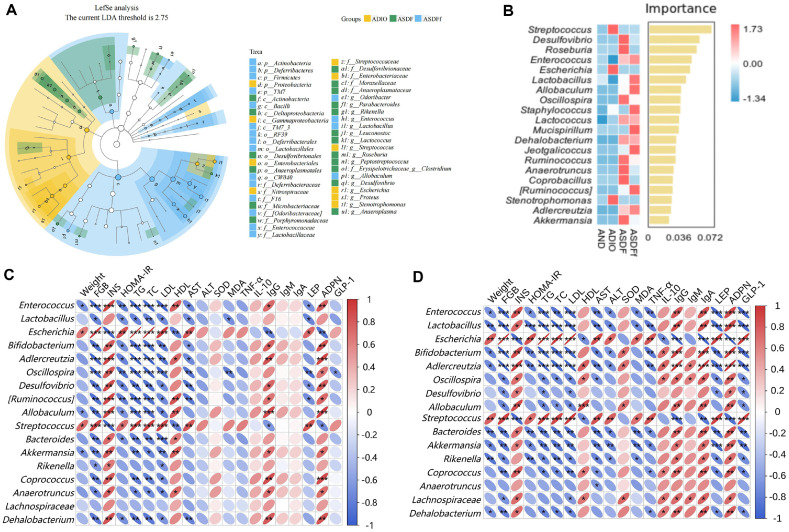
Marker species taxonomic LefSe (LDA Effect Size) analysis (A). Node size corresponds to the mean relative abundance of the OTU; hollow nodes represent OTUs with non-significant differences between groups, while solid nodes represent OTUs with higher abundance and significant differences between groups. Letters identify the names of taxonomic units with significant differences between groups; Random forest model analysis of genus level marker species (**B**). Importance on the right side indicates that from top to bottom species are of decreasing importance to the model, and it can be assumed that these species at the top of the importance scale are marker species for differences between groups; Spearman's correlation analysis between intestinal flora and obesity indicators between ADIO vs ASDF groups (**C**) *P < 0.05, **P < 0.01, ***P < 0.001), and ADIO vs ASDFf groups (**D**) *P < 0.05, **P < 0.01, ***P < 0.001) (*n* = 6).

This study further investigated the effect of G-SDF on intestinal marker species and their relationship with pathological indicators of ADIO mice using LEfSe, random forest, and Spearman correlation analyses. LEfSe analysis (Fig. 7A) revealed that marker species in the ADIO group were concentrated in Proteobacteria, while those in the ASDF group shifted to Actinobacteria. In the ASDFf group, marker species expanded to Firmicutes, Deferribacteres, TM7 further. Random forest analysis (Fig. 7B) indicated that *Streptococcus* and *Escherichia* were high-abundance marker species in the ADIO group, *Desulfovibrio*, *Roseburia*, and *Enterococcus* were predominant in the ASDF group, and *Enterococcus*, *Lactobacillus*, and *Allobaculum* were marker species in the ASDFf group.

Spearman’s correlation analysis were used to examine the relationship between intestinal flora structure and body weight, blood glucose (FGB, INS, and HOMA-IR), lipids (TC, TG, LDL, and HDL), liver function (ALT and AST), oxidative stress (SOD and MDA), inflammatory factors (TNF-α and IL-10), immune factors (IgG, IgA, and IgM), and fat metabolism factors (LEP, ADPN, and GLP-1). As shown in Figure 7C, *Enterococcus*, *Allobaculum*, and *Akkermansia* were significantly negatively correlated with body weight, while *Escherichia* and *Streptococcus* showed a significant positive correlation (*P* <0.05). *Enterococcus*, *Lactobacillus*, *Bifidobacterium*, *Adlercreutzia*, *Oscillospira*, *Desulfovibrio*, *Ruminococcus*, *Bacteroides*, *Akkermansia*, *Coprococcus*, *Anaerotruncus*, and *Dehalobacterium* were significantly positively correlated with blood glucose (FGB and HOMA-IR), lipids (TC, TG, and LDL), AST, and IgG, and significantly negatively correlated with INS, HDL, and ADPN (*P* < 0.05), while *Escherichia* and *Streptococcus* showed opposite correlations. Additionally, *Lactobacillus* showed a significant negative correlation with MDA (*P* < 0.05), and *Oscillospira* was significantly negatively correlated with MDA (*P* < 0.01) and GLP-1 (*P* < 0.05). As shown in Fig. 7D, a similar correlation trend was observed in the ASDFf group. Additionally, *Anaerotruncus* showed a significant negative correlation with AST in the ASDFf group (*P* < 0.05). *Enterococcus*, *Lactobacillus*, *Bifidobacterium*, *Adlercreutzia*, *Bacteroides*, *Akkermansia*, *Coprococcus*, and *Dehalobacterium* were negatively correlated with the pro-inflammatory factor TNF-α and positively correlated with the anti-inflammatory factor IL-10, while *Escherichia* and *Streptococcus* showed opposite correlations (*P* <0.05).

### Effect of G-SDF and SDFfbs on the Intestinal Flora Function

**Fig. 8:**
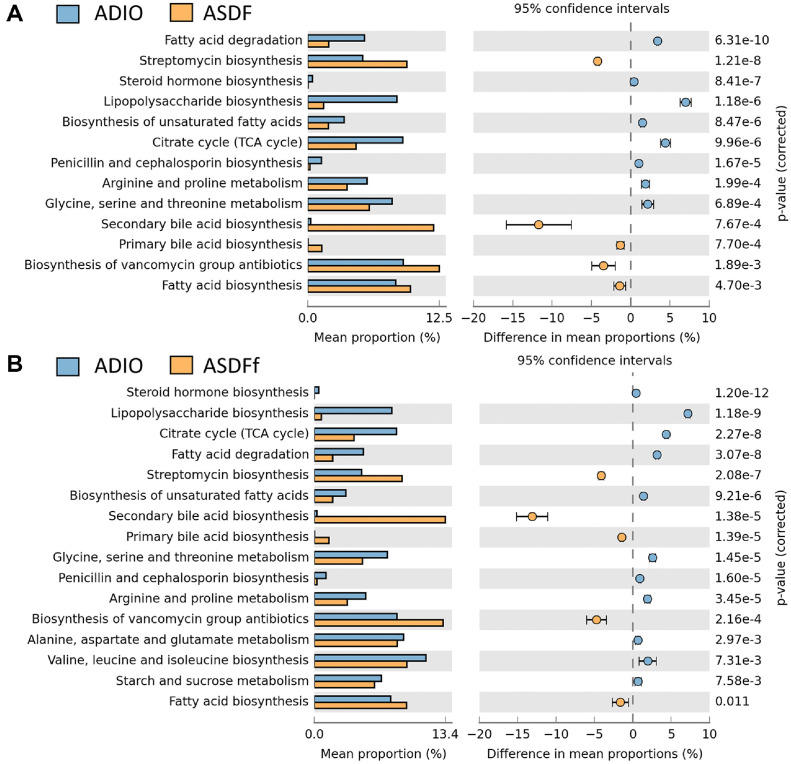
Metabolic pathways gene changes of intestinal flora between ADIO vs ASDF groups (A) and ADIO vs ASDFf groups (B) (*n* = 6).

KEGG pathway analysis was conducted by using PICRUSt2 to prediction function changes of intestinal flora. Results indicated that the gene abundance of primary signaling pathways were similar among groups (Fig. S2). Among secondary signaling pathways, the gene abundance of metabolism-related pathways varied significantly. Specific pathways related to obesity and antibiotic were selected for detailed analysis. As shown in Figure 8, compared to the ADIO group, the ASDF group significantly upregulated the gene abundance involved in streptomycin biosynthesis, secondary bile acid biosynthesis, primary bile acid biosynthesis, and fatty acid biosynthesis. Conversely, the gene abundance significantly downregulated in pathways involved in fatty acid degradation, steroid hormone biosynthesis, LPS biosynthesis, biosynthesis of unsaturated fatty acids, the citrate cycle (TCA cycle), penicillin and cephalosporin biosynthesis, arginine and proline metabolism, and glycine, serine, and threonine metabolism in the ASDF group. The ASDFf group exhibited a similar trend to the ASDF group. Additionally, the ASDFf group significantly reduced the gene abundance involved in steroid biosynthesis, biosynthesis of vancomycin group antibiotics, alanine, aspartate, and glutamate metabolism, valine, leucine, and isoleucine biosynthesis, and starch and sucrose metabolism (*P* < 0.05).

## Discussion

In recent decades, antibiotics have been widely used in the clinic and for livestock and poultry breeding because of their strong bacteriostatic and growth-promoting properties. However, their use also poses risks, such as gut dysbiosis and immune system impairment, in both animals and humans. Studies have shown that gut microbiota significantly influence obesity development by regulating energy balance, insulin resistance, and inflammatory responses, which play a central role in obesity and related metabolic diseases. In this study, we found that both G-SDF and SDFfbs effectively reduced HFD-induced increases in body weight, blood lipids, liver fat, and insulin resistance, even under antibiotic interference. Additionally, both treatments significantly improved antibiotic-induced cecal edema and colonic villus damage and alleviated obesity-induced hepatic impairment. These findings suggest that G-SDF and SDFfbs have multiple effects on weight loss, fat reduction and resistance to antibiotic interference.

Antibiotics exacerbate the alterations in gut microbiota composition induced by HFD, and facilitate bacterial infiltration of the gut lining [16]. Additionally, HFD and antibiotics synergistically impair mitochondrial function, leads to oxygen leakage into the gut, disrupts the balance among intestinal flora [17, 18]. Occludin and Claudin1, members of the Claudin protein family, are crucial for maintaining the selective permeability of epithelial cells and for barrier integrity [19]. This study revealed that both G-SDF and SDFfbs significantly increased Occludin and Claudin1 expression in colon tissues, thereby helping to maintain intestinal barrier integrity. The LPS/TLR4/MyD88/NF-κB signaling pathway is a key inflammatory response pathway in the immune system. TLR4, a pattern recognition receptor on the cell surface, binds to ligands such as LPS, recruits MyD88, and initiates downstream signaling. MyD88, a key adapter protein, facilitates the phosphorylation and degradation of IκBa via interaction with the IκB kinase complex, thereby releasing the NF-κB p65 and p50 subunits, which then translocate to the nucleus to activate the expression of inflammation-related genes [20]. Our study demonstrated that G-SDF and SDFfbs significantly reduced the protein expression of the LPS/TLR4/MyD88/NF-κB pathway, mitigated ABX-induced inflammation, and preserved intestinal barrier integrity. In particular, SDFfbs maintained the intestinal protective effects of G-SDF despite ABX interference, which suggested that FMT could be an effective method to deliver SDF and harness its effects.

Advances in sequencing technologies have improved the understanding of the complex link between gut microbiota and obesity. Research has shown that HFD compromises mitochondrial function in intestinal epithelial cells, increases Firmicutes populations, promotes the proliferation of harmful *Enterobacteriaceae*, and reduces the abundance of Bacteroidetes [21]. An increased abundance of Proteobacteria may indicate gut dysbiosis and increased disease risk. Firmicutes represent the most active bacterial group in individuals with severe obesity, and an abnormally high Firmicutes/Bacteroidetes (F/B) ratio is generally associated with obesity [21, 22]. This study revealed that antibiotics significantly reduced the intestinal flora diversity of the AND and ADIO groups, in which Proteobacteria predominated and Bacteroidetes nearly vanished. In contrast, treatment with G-SDF and SDFfbs significantly increased the abundance of Firmicutes, Actinobacteria, and Bacteroidetes, substantially decreased the abundance of Proteobacteria. These findings indicate that antibiotic administration severely damaged the native flora of obese mice and increased the abundance of pathogenic Proteobacteria. In contrast, G-SDF and SDFfbs partially countered the effects of antibiotics.

Previous animal studies have shown that a HFD does not lead to an abnormal increase in *Escherichia* in the intestines of mice; rather, ABX intervention may increase the abundance of *Escherichia* [23-25]. This study revealed that the abundance of *Escherichia* increased from 0.01654% in the AND group to 28.15% in the ADIO group due to ABX interference, which shows that the combined effect of ABX and HFD significantly exacerbates intestinal flora damage in mice. Moreover, *Lactobacillus*, *Enterococcus*, *Bifidobacterium*, *Adlercreutzia*, *Oscillospira*,[*Ruminococcus*], *Allobaculum*, and *Bacteroides* were significantly enriched in the ASDF and ASDFf groups. Research has shown that red ginseng dietary fiber increases the abundance of *Lactobacillus* and decreases body weight in obese mice [26]. *Lactobacillus* can also lower the intestinal pH by producing lactic acid and other short-chain fatty acids (SCFAs), such as butyric acid, thereby inhibiting the growth of pathogenic bacteria, maintaining the intestinal microbiota balance, and reducing the impact of antibiotics on the intestinal flora of obese mice [27, 28]. *Lactobacillus* also participates in the metabolism of polyunsaturated fatty acids (PUFAs), which produce intermediates such as hydroxy fatty acids, oxo fatty acids, conjugated fatty acids, and partially saturated trans fatty acids, which contribute to fatty acid degradation and metabolism [29-31]. Gavage of ginseng extract (GE) in obese mice increased the abundance of *Enterococcus*, which metabolizes the long-chain fatty acid myristoleic acid (MA)[8]. This product activated UCP1 and promoted H^+^ transfer across the mitochondrial membrane, which then activated brown fat thermogenesis and promoted weight loss [32]. Clinically, *Enterococcus granulosus* functions as an antioxidant and is resistant to several antibiotics, including vancomycin, penicillin, erythromycin, high-level gentamicin, and tetracycline [33]. Additionally, *Enterococcus* can metabolize spermidine in the intestinal tract and convert it into spermine via arginine decarboxylase activity [34]. This study revealed a significant increase in the abundance of *Enterococcus*, an expansion of the brown fat area, and reduced expression of genes in the arginine and proline metabolism pathways in the ASDF and ASDFf groups (*P* < 0.05), which suggested that the regulatory effects of G-SDF and SDFfbs on *Enterococcus* may indirectly regulate the metabolism of these amino acids. *Bifidobacterium* breaks down carbohydrates (such as lactose) in the intestine into lactic acid and acetic acid through glycolysis [35]. Lactic acid can effectively inhibit the growth of spoilage bacteria and harmful bacteria, whereas acetic acid can promote intestinal peristalsis and the secretion of immunoglobulin IgA, activate macrophages and NK cells, and produce cytokines, such as IL-1, IL-6, IL-12, TNF-α, and IFN-γ, thereby regulating the body's immune response [36]. In addition, the extracellular polysaccharide (EPS) produced by *Bifidobacterium* has immunomodulatory properties and can reduce the inflammatory response induced by LPS [37,38]. This study revealed a significant increase in the abundance of *Bifidobacterium* in the ASDF and ASDFf groups, along with a notable decrease in the expression of genes related to the LPS biosynthesis pathway (*P* <0.05), which suggested that the beneficial effects of G-SDF and SDFfbs on LPS biosynthesis may be facilitated by their regulation on *Bifidobacterium*.

*Parabacteroides* were significantly enriched and exclusively present in the ASDF group in this study (Fig. S1A). Inulin dietary fiber was confirmed to increase the abundance of *Parabacteroides* in obese mice and protect mice from nonalcoholic fatty liver disease (NASH) [39]. Inulin reduces TLR4 signaling and Akt activation, thereby inhibiting colon tumorigenesis in AOM-treated mice fed a HFD [40]. Additionally, *Parabacteroides* can also convert primary bile acids to secondary bile acids, such as lithocholic acid and ursodeoxycholic acid, which support lipid metabolism by activating the intestinal FXR signaling pathway [41]. This study revealed a marked increase in TLR4 protein expression in the ASDF group, alongside an increase in the expression of genes related to the secondary bile acid production pathway in the gut microbiota, which was likely associated with a substantial increase in *Parabacteroides* abundance. Furthermore, this study revealed a significant increase in the abundance of *Akkermansia* in the ASDF group (Fig. S1C). SDF from grapefruit peel and ginseng extract (containing saponins, polysaccharides) also significantly increased the abundance of *Akkermansia*, regulated lipid metabolism, and mitigated HFD-induced obesity [42]. *Akkermansia* has been recognized for its role in promoting gut barrier integrity by increasing mucus layer thickness; increasing the expression of tight junction proteins (*e.g.*, ZO-1), antimicrobial peptides, and immunomodulatory factors; and facilitating the synthesis of SCFAs (*e.g.*, butyric acid and propionic acid) and hydrogen sulfide, thereby reducing inflammation and adiposity [43, 44]. Additionally, *Akkermansia* promotes insulin release by increasing the secretion of GLP-1, which subsequently increases insulin sensitivity and glucose tolerance [45].

A Spearman correlation analysis (Fig. 7) revealed that *Enterococcus*, *Lactobacillus*, *Bifidobacterium*, *Adlercreutzia*, *Oscillospira*, *Desulfovibrio*, *Ruminococcus*, *Bacteroides*, *Akkermansia*, *Coprococcus*, *Anaerotruncus*, and *Dehalobacterium* were strongly and positively correlated with blood glucose and lipid levels. Additionally, *Enterococcus*, *Lactobacillus*, *Bifidobacterium*, *Adlercreutzia*, *Bacteroides*, *Akkermansia*, *Coprococcus*, and *Dehalobacterium* were negatively associated with proinflammatory factors and positively correlated with anti-inflammatory factors. These findings suggest that G-SDF may improve glycolipid metabolism markers in obese mice by modulating the composition of certain intestinal flora, potentially counteracting the combined adverse effects of antibiotic exposure and obesity.

Further investigations into metabolic pathways revealed that G-SDF and SDFfbs may target the tricarboxylic acid (TCA) cycle and LPS production (Fig. 8). The TCA cycle functions as a central hub for the metabolic integration of three primary nutrients: carbohydrates, lipids, and amino acids. It generates energy through the complete oxidative degradation of carbohydrates and other compounds. The hepatic TCA cycle, which involves terminal fat oxidation and gluconeogenesis, is upregulated in obese mice, with increased metabolites detected in white adipose tissue. LPS, a major pathogenic component in bacterial infections, triggers symptoms such as fever, leukocyte activation, and disruptions in glucose metabolism [46]. This study demonstrated that G-SDF and SDFfbs inhibited the LPS/TLR4/MyD88/NF-κB signaling pathway while simultaneously reducing the expression of genes related to the TCA cycle and LPS biosynthesis. This finding may explain the mechanism by which G-SDF and SDFfbs mitigate nutrient absorption and inflammation in obese mice with intestinal disorders.

Bile acids are the main components of bile that perform the functions of digestion and absorption of lipids and the excretion of specific metabolites (*e.g.*, cholesterol and bilirubin) in feces [47, 48]. Research has shown an accumulation of primary bound bile acids in germ-free (GF) and antibiotic-treated mice, indicating that intestinal flora plays a crucial role in bile acid metabolism [49]. Individuals with intestinal disorders, such as inflammatory bowel disease (IBD), commonly exhibit dysregulated gut ecology characterized by reduced the intestinal flora diversity and the Firmicutes abundance. These changes result in decreased levels of secondary bile acids and elevated levels of conjugated bile acids in the gut [50]. This study revealed that the expression levels of genes related to primary and secondary bile acid synthesis pathways were significantly lower in the ADIO group than in the ASDF and ASDf groups. These findings suggest that G-SDF and SDFfbs may promote weight reduction in obese mice with gut dysbiosis by modulating bile acid synthesis.

## Conclusion

This study demonstrated that G-SDF promoted weight loss and antibiotic inhibition in gut dysbiosis obese mice. Specifically, antibiotic treatment exacerbated abnormality in glycolipid metabolism, oxidative stress, and inflammatory markers in obese mice, whereas G-SDF and SDFfbs effectively reduced the above symptoms, inhibited the LPS/TLR4/MyD88/NF-κB signaling pathway in ADIO mice. G-SDF and SDFfbs also restored ABX-induced gut dysbiosis and preserved intestinal barrier integrity. Furthermore, we found that both G-SDF and SDFfbs effectively regulated TCA cycle activity and improved bile acid synthesis in ADIO mice. Our findings indicate that SDFfbs can replicate the weight loss efficacy of G-SDF and even provide additional benefits, suggesting that fecal transplantation may represent a novel promising intervention for weight loss, which, together with the use of G-SDF, could be an alternative to conventional therapies. In conclusion, this study revealed that G-SDF and SDFfbs can promote weight loss, reduce lipid accumulation, strengthen the intestinal barrier, inhibit the LPS/TLR4/MyD88/NF-κB signaling pathway through the modulation of the intestinal flora, and resist the double damage caused by obesity and antibiotics. This study provides a critical theoretical foundation for the wide application of G-SDF, including its potential in preventing obesity and antibiotic resistance linked to HFD and gut dysbiosis, and the application value of its new intervention method of fecal bacteria transplantation.

## Supplemental Materials

Supplementary data for this paper are available on-line only at http://jmb.or.kr.



## Figures and Tables

**Table 1:** Mice blood lipid and liver lipid index assays.