# Supplementary material for: Protective Effects of Ginseng Soluble Dietary Fiber and Its Fecal Microbiota Extract on Antibiotic-Induced Gut Dysbiosis Obese Mice
Source: J Microbiol Biotechnol. 2025 Jul 14;35:e2502013. doi: 10.4014/jmb.2502.02013 (PMC12283264; doi:10.4014/jmb.2502.02013)
Supplement: Supplementary file 1 [file jmb-35-e2502013-supple.pdf]

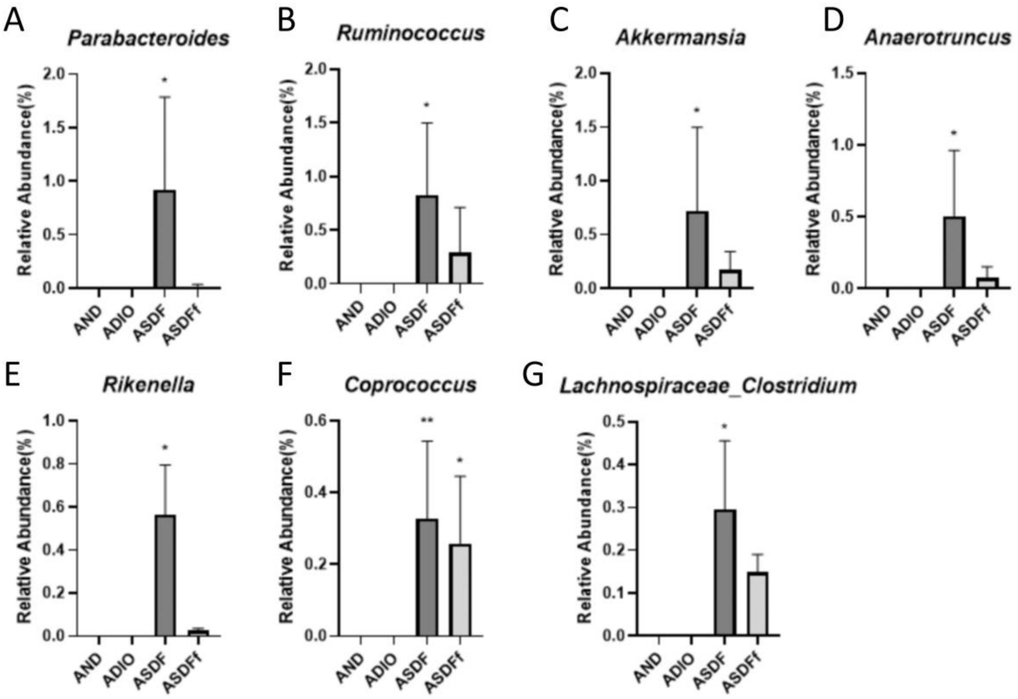

Fig. S1 Abundance of intestinal flora showing significant differences at the genus level, with an average abundance below 1% (n = 6). Compared with the ADIO group, * *P*<0.05, ** *P*<0.01.

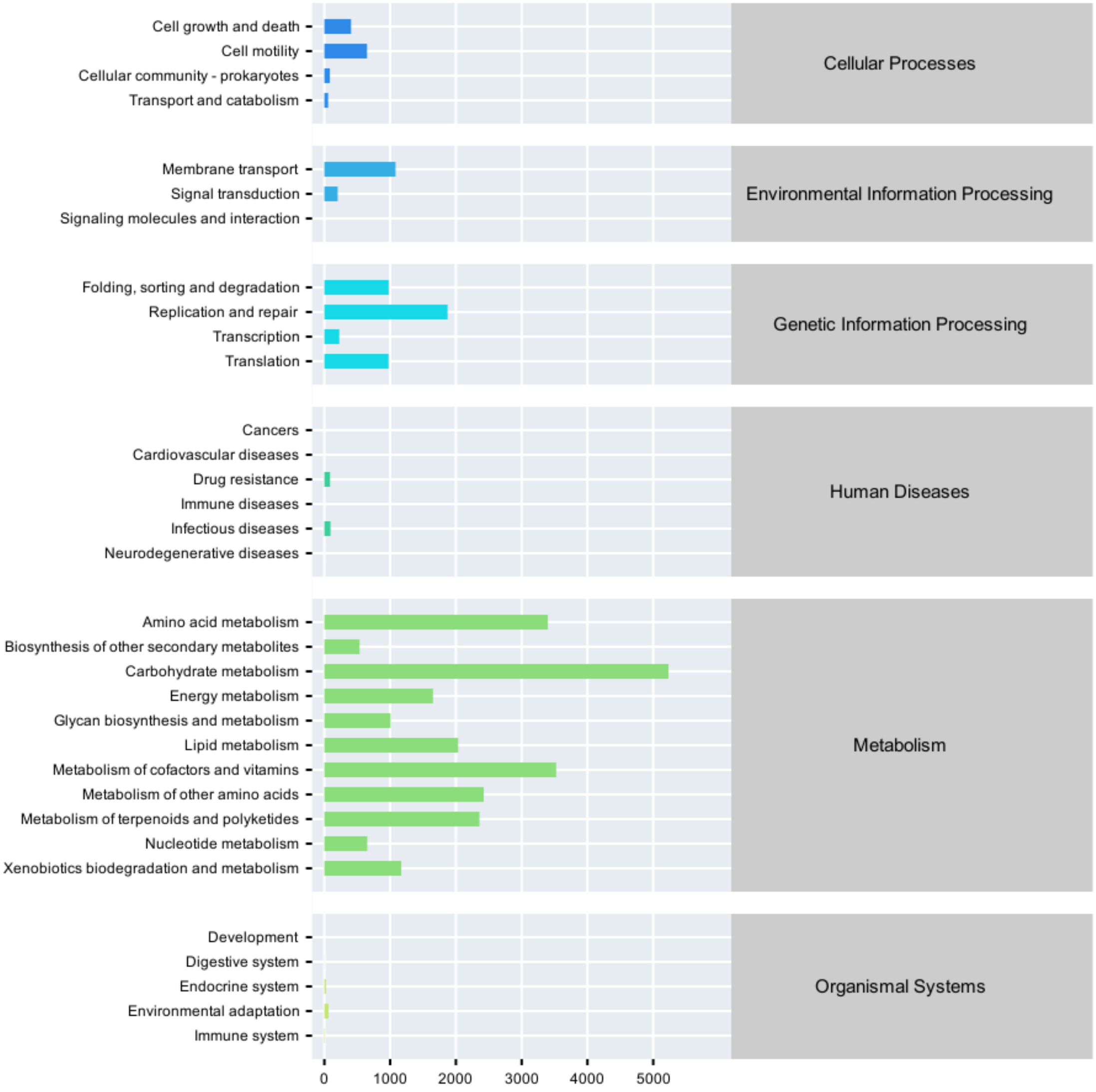

Fig. S2 Relative abundance of KEGG signaling pathway functional gene expression in all four groups.

Table S1. Chemical composition of G-SDF and viable bacteria count of SDFfbs

Group	Nutrition	%/(CFU/mL)	Monosaccharide	mol%
G-SDF	Water	12.76±0.13	Mannose	1.94±0.01
	Protein	13.16±0.11	D-glucosamine	3.07±0.02
	Fat	0.15±0.05	Rhamnose	0.47±0.02
	Ash	6.17±0.43	Glucuronic acid	0.17±0.01
	Carbohydrate	72.89±0.64	Galacturonic acid	2.74±0.02
	Total Sugar	63.54±0.87	Glucose	61.13±0.64
	Uronic Acid	5.04±0.33	Galactose	16.54±0.02
	Total Saponin	0	Xylose	12.67±0.18
			Trehalose	7.99±0.08
SDFfbs	Viable count	$3.14 \pm 0.12 \times 10^7$		

Table S2. Organ index of mice (%).

	Liver/Body Weight	eWAT/Body Weight	iWAT/Body Weight	BAT/Body weight
AND	3.69±0.13[a]	1.03±0.09[a]	0.27±0.09[a]	0.25±0.01[c]
ADIO	3.42±0.09[a]	2.67±0.24[c]	1.52±0.55[c]	0.10±0.02[a]
ASDF	3.32±0.10[a]	2.32±0.48[bc]	1.32±0.16[bc]	0.14±0.02[b]
ASDFf	3.34±0.11[a]	2.05±0.34[b]	1.13±0.23[b]	0.17±0.02[bc]

Different lowercase letter shoulder marks mean significant differences ($P<0.05$).
